# STN–ANT plasticity is crucial for the motor control in Parkinson’s disease model

**DOI:** 10.1038/s41392-021-00545-z

**Published:** 2021-06-09

**Authors:** Hui Zhang, Chunkui Zhang, Zhongwei Qu, Bing Li, Yujuan Su, Xia Li, Yan Gao, Yizheng Wang

**Affiliations:** 1 Center of Cognition and Brain Science, Beijing Institute of Basic Medical Sciences, Beijing, China; 2 Laboratory of Neural Signal Transduction, Institute of Neuroscience, Chinese Academy of Sciences, Shanghai, China; 3 National Clinical Research Center for Aging and Medicine, Huashan Hospital, Fudan University, Shanghai, China

**Keywords:** Diseases of the nervous system, Molecular neuroscience, Cellular neuroscience

**Dear Editor**,

Motor control as a function of the basal ganglia circuit is crucial for every aspect of life and movement disorders, such as Parkinson’s disease (PD). In PD, the progressive denervation of dopamine in the dorsal striatum leads to inhibition of the direct pathway and facilitation of the indirect pathway and results in activation of the subthalamic nucleus (STN) and globus pallidus internus (GPi), two important nuclei in the motor loop of basal ganglia.^1^ Indeed, manipulating STN or GPi via deep brain stimulation (DBS) can correct motor symptoms of both PD patients and animal models. STN-DBS greatly suppresses the resting tremor and reduces dopaminergic medications, but increases the risk of falls, whereas GPi-DBS mainly benefits dyskinesia and gait.^2^ These observations suggest that other circuit in addition to STN–GPi circuit also plays a role in motor control.

We initially mapped the STN-projecting nuclei with a series of viral tracing studies. First, adeno-associated virus (AAV) expressing enhanced yellow fluorescent protein (EYFP) was injected into the unilateral mouse STN (Supplementary Fig. S1a, b). Besides the brain areas well known to receive STN projections, like globus pallidus externa (GPe) and GPi (Supplementary Fig. S1c), the EYFP-positive fibers were also evident in the anterior thalamic nucleus (ANT) (Fig. 1a), a nucleus known to project to cingulate cortex and important for voluntary movement. To further examine this previously unidentified STN–ANT circuit, we injected trans-monosynaptic AAV expressing Cre recombinase or AAV harboring Dio-EYFP into STN or ANT, respectively, (Supplementary Fig. S1d, top and middle, left) to mark the ANT neurons that receive projections from STN. The EYFP-positive cells in ANT were co-labeled with NeuN, a neuronal marker (Supplementary Fig. S1d, middle, right). Further analysis revealed that most of the EYFP-positive neurons were excitatory (Fig. 1b and Supplementary Fig. S1d, bottom, left). Moreover, retrograde viral tracing experiments also confirmed the STN–ANT connection (Supplementary Fig. S1e). Next, whether the STN–ANT circuit is functionally monosynaptic was tested by optogenetics and whole-cell recording (Fig. 1c, left). Optical stimulation of STN-projecting fibers on ANT slice evoked excitatory postsynaptic currents (EPSCs) on ANT neurons, with an average latency of 4.81 ± 0.34 ms and amplitude of 50.27 ± 13.69 pA (Supplementary Fig. S1f). The evoked EPSCs were sensitive to CNQX (Supplementary Fig. S1f, right), but insensitive to tetrodotoxin and 4-aminopyridine (Fig. 1c, right). Altogether, these results suggested an undiscovered, monosynaptic, and excitatory projection from STN to ANT.

**Fig. 1:**
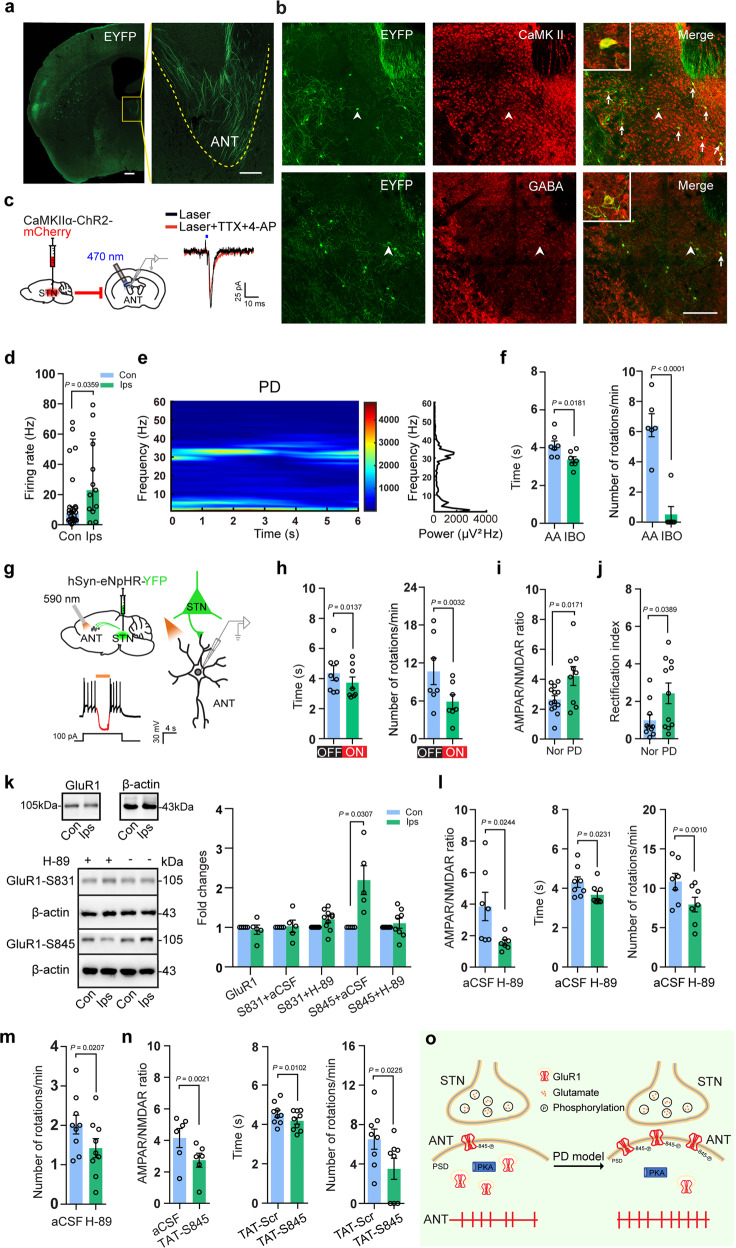
STN–ANT plasticity is crucial for the motor control in Parkinson’s disease model. **a** Representative images of EYFP-positive fibers in ipsilateral ANT in coronal brain slices. *n* = 5 mice. Scale bar, 200 μm. **b** Representative images of EYFP-positive ANT cells stained with anti-CaMKII or -GABA antibodies. Short arrows pointed to the neurons co-labeled. Inset: magnified views of arrowhead regions. Scale bar, 200 μm. *n* = 5 mice. **c** Schematics for in vitro examination of the functional connection from STN to ANT (left). Representative trace of the evoked EPSC in ANT neurons in the presence of TTX and 4-AP (right). **d** Firing rate of neurons in contralateral and ipsilateral ANT of PD model rats. *n* = 11 rats. **e** Spectrogram and power spectrum density analysis of local field potentials in PD model rats. **f** Time of passing the beam and the number of APO-induced rotations in PD model mice with injection of solvent control (ascorbic acid, AA) or IBO in ipsilateral ANT (right). *n* = 6–7 mice in each group. **g** Schematics of optical stimulation (yellow bar, 590 nm, 4 s, continuous) of eNpHR-positive fibers from STN in ipsilateral ANT (top) and functional verification of eNpHR (bottom). *n* = 3 mice. **h** Time of passing the beam and the number of APO-induced rotations in PD model mice with the 590 nm optical stimulation on or off. *n* = 7–8 mice in each group. **i** AMPAR/NMDAR current ratio evoked by optical stimulation. *n* = 6–7 mice in each group. **j** Rectification index of AMPAR stimulated by optical stimulation in normal (Nor) and PD model mice. *n* = 6–7 mice in each group. **k** Western blot images and quantitative analysis of GluR1, GluR1-S831, H-89/GluR1-S831, GluR1-S845, H-89/GluR1-S845 using the extracts from contralateral (Con) or ipsilateral (Ips) ANTs of PD mice. *n* = 5–10 mice in each group. β-actin was used as a loading control. **l** AMPAR/NMDAR current ratio in ipsilateral ANT neurons evoked by electrical stimulation in the presence of aCSF or H-89 (left) (*n* = 3 mice) and time of passing the beam and the number of APO-induced rotations in PD model mice after infusion of H-89 into ANT (middle and right). *n* = 7–8 mice in each group for the behavioral test. **m** The number of APO-induced rotations in MPTP-PD model mice after infusion of H-89 into ANT. *n* = 9 mice. **n** AMPAR/NMDAR current ratio in ipsilateral ANT neurons in the presence of aCSF or TAT-S845 in PD model mice (left) (*n* = 3 mice) and time of passing the beam and the number of APO-induced rotations in PD model mice after infusion of TAT-S845 into ANT (middle and right). *n* = 7–8 mice in each group for the behavioral test. **o** Schematic shows the strengthen of synaptic plasticity in STN–ANT circuit resulted from increased PKA phosphorylation of GluR1-S845 in PD models and which in turn play a critical role in PD motor deficits. In **d**, **i**, **j**, **l** (left), **n** (left) each circle represents a neuron, and in **f**, **h**, **k**, **l** (middle and right), **m**, **n** (middle and right) each circle represents a mouse. Mann–Whitney test was used for analysis of firing rates from in vivo recording. The other data were analyzed by Student’s *t* test. Data are presented as mean ± SEM of at least three independent experiments, except firing rates from in vivo recording (**d**) which were presented as median (interquartile range)

Since the activity of excitatory STN neurons was increased in PD^1^ and STN-projecting ANT neurons are mainly excitatory, we wondered whether the activity of ipsilateral ANT was increased in PD model rodents. We established hemiparkinsonian rodent models by injecting 6-OHDA into the unilateral dorsal striatum (mice) or medial forebrain bundle (MFB) (rats) to induce a distinct loss of dopamine neurons in unilateral substantia nigra compacta (SNc) (Supplementary Fig. S2a). In hemiparkinsonian model mice, c-fos expression was clearly enhanced in ipsilateral, but not in contralateral ANT. While lesion of ipsilateral STN by ibotenic acid (IBO) reversed the enhanced c-fos expression (Supplementary Fig. S2b–d). To characterize this increased activity in vivo, we then did multichannel electrophysiological recordings in freely behaving rats (Supplementary Fig. S2e). The firing rate of the neurons in the ipsilateral ANT was greatly increased than that in the contralateral ANT of PD model rats (Fig. 1d). A distinct band of local field potential (LFP) activity in the beta range (15–35 Hz) (Supplementary Fig. S2f, g), regarded as a pathophysiological marker of PD motor deficits,^3^ was observed in ipsilateral ANT. The total power of LFP between 15–35 Hz was markedly increased in five out of nine PD model rats (Fig. 1e) compared with that in five normal rats.

To investigate the role of enhanced neural activity in STN–ANT in motor control of PD model mice (Supplementary Fig. S3a), we applied a balance beam test and apomorphine (APO)-induced rotations (Supplementary Fig. S3b). Lesion of ANT by IBO in PD model mice dramatically improved motor performance with a decrease of time for passing through the beam and the number of contralateral rotations (Fig. 1f). Consistently, suppressing ANT (Supplementary Fig. S3c, d) or STN–ANT circuit via optogenetics ameliorated the motor deficits (Fig. 1g, h and Supplementary Fig. S3e, f).

We next studied how the enhanced STN–ANT neural activity leads to chronic motor abnormalities in PD model mice. As ANT neurons received glutamatergic inputs from STN and glutamate receptors are important for the establishment and maintenance of synaptic activity, we initially examined the activities of AMPAR and NMDAR, two important glutamate receptors, in ANT and STN–ANT circuit. The AMPAR/NMDAR current ratio was largely increased both in ANT and STN–ANT circuit (Supplementary Fig. S4a, b and Fig. 1i), indicating that the excitatory synaptic transmission was greatly enhanced in STN–ANT circuit of PD model mice. It is known that the increased expression of GluR2-lacking AMPARs (GluR1 is the primary component) on the postsynaptic membrane is essential for long-term synaptic potentiation.^4^ Indeed, the rectification index of AMPARs, reflecting the GluR2-lacking AMPARs on the membrane, both in ANT and STN-projecting ANT neurons of PD model mice were markedly increased (Supplementary Fig. S4c and Fig. 1j), indicating the accumulation of GluR2-lacking AMPARs on the membrane of the ipsilateral ANT neurons in PD model mice. Furthermore, previous reports suggested that GluR1 membrane expression is regulated by phosphorylation of its intracellular carboxy-terminal motif.^5^ The phosphorylation sites usually include serine 831 (S831), phosphorylated by CaMKII or protein kinase C, and serine 845 (S845), phosphorylated by protein kinase A (PKA). Using phosphorylation site-specific antibodies, we found that GluR1-S845, but not GluR1-S831, was greatly increased in ipsilateral ANT, whereas the expression of total AMPAR-GluR1 was not changed. Applying H-89, a specific PKA inhibitor, into the ipsilateral ANT or ANT slice blocked the increased GluR1-S845 phosphorylation (Fig. 1k), brought the increased AMPAR/NMDAR ratio to the control levels (Fig. 1l, left and Supplementary Fig. S4d). Moreover, delivery of H-89 into ipsilateral ANT alleviated motor deficits of both 6-OHDA-treated mice (Fig. 1l, middle and right, Supplementary Fig. S4e) and MPTP-treated mice (Fig. 1m). To specifically prevent the phosphorylation of GluR1-S845, we designed TAT-S845, a cell-permeable peptide that contained a sequence spanning the PKA phosphorylation site in GluR1 (Supplementary Fig. S4f). Application of TAT-S845 reversed the increase of AMPAR/NMDAR current ratio to the control levels and alleviated motor deficits of PD model mice (Fig. 1n).

Together, the present experiments showed that the synaptic plasticity of STN–ANT circuit controls the motor behaviors in PD model rodents (Fig. 1o). The newly identified and characterized STN–ANT circuit may represent a circuit that specifically relays motor signals from the basal ganglia (STN) to the cingulate cortex for sensory-motor integration and synaptic plasticity in this circuit participates in the regulation of the motor deficits in the PD models. Dissecting the role of STN–ANT circuit will provide new precise targets for neuronal modulations. It is not clear at present why the synaptic plasticity changes were observed in STN–ANT, but not in STN–GPi circuit (Supplementary Fig. S4g). In summary, the current findings provided at a comprehensive level (circuit, synaptic, and molecular) a clear dissection that synaptic plasticity regulated motor control.

## Supplementary information

supplementary information

Supplementary material-SIGTRANS-0230R1

supplementary Figure1a-f

supplementary Figure2a-g

supplementary Figure3a-f

supplementary Figure4a-g

Supplementary information

## Data Availability

The datasets in this study are available from the corresponding author upon reasonable request.
