# Supplementary material for: STN–ANT plasticity is crucial for the motor control in Parkinson’s disease model
Source: Signal Transduct Target Ther. 2021 Jun 9;6:215. doi: 10.1038/s41392-021-00545-z (PMC8187716; doi:10.1038/s41392-021-00545-z)
Supplement: Supplementary file 1 — supplementary information [file 41392_2021_545_MOESM1_ESM.docx]

Supplementary Materials for

STN-ANT plasticity is crucial for the motor control in Parkinson's disease model

Hui Zhang^1^, Chunkui Zhang^1^, Zhongwei Qu^2^, Bing Li^1^, Yujuan Su^1^, Xia Li^1^, Yan Gao^1^ and Yizheng Wang^1,3,^*

1 Center of Cognition and Brain Science, Beijing Institute of Medical Sciences, Beijing, 100850, China.

2 Laboratory of Neural Signal Transduction, Institute of Neuroscience, Chinese Academy of Sciences, Shanghai, 200040, China.

3 National Clinical Research Center for Aging and Medicine, Huashan Hospital, Fudan University, 12 Middle Wulumuqi Road, Shanghai 200040, China.

These authors contributed equally: Hui Zhang, Chunkui Zhang, Zhongwei Qu.

*Correspondence to: Yizheng Wang (yzwang@ion.ac.cn)

**This PDF file includes:**

Materials and Methods

Fig.S1 to S4

References

**Materials and methods**

**Animals**

Male C57/BL6 mice (5-8 weeks old) and Sprague-Dawley (SD) rats (250-350 g) were housed up to five mice or two rats per cage with a 12 hours light-dark cycle at constant temperature (21℃) and humidity. The access to get food and water were *ad libitum*. All procedures for the care and use were carried out in accordance with Institute of Cognition and Brain Sciences guidelines and all efforts were made to ameliorate animals suffer.

**Establishment of hemi-parkinsonian rodent models with 6-OHDA**

The hemi-parkinsonian rodent models were generated following the methods as described previously^1,2^. Briefly, desipramine (25 mg/kg for mice, 40mg/kg for rats, Sigma, D3900) was intraperitoneally injected into the rodents. After 30 min, 6-OHDA (2μg ul^-1^ and 2 μl for mice, 5 μg μl^-1^ and 3 μl for rats, dissolved in 0.2 % (w/v) ascorbic acid (AA), Sigma, H4381) was injected into left striatum (for mice) or one side of MFB (for rats) according to the stereotaxic coordinates (left striatum, AP: 0.4 mm, ML: 1.8 mm, DV: -3.5 mm; MFB, AP: -4.3 mm, ML: ±1.5 mm, DV: -7.6 mm) with a Hamilton syringe (5μl ,7633-01) controlled by a syringe pump (KD scientific, 788130) at a rate of 0.1 μl min^-1^. After injection, the syringe was left in place for 5 min to avoid backflow. Two weeks (for mice) or three weeks (for rats) after 6-OHDA injection, apomorphine (0.5 mg kg^-1^, Sigma, A4393)-induced rotation test was conducted to evaluate whether the establishment of hemi-parkinsonian rodent models were successful. Rotation numbers were counted manually 3 min after apomorphine injection and rodents exhibiting more than 40 rotations in 10 min were chosen in the following studies. After all experiments, loss of dopaminergic neurons in SNc was evaluated with immunostaining for tyrosine hydroxylase.

**Establishment of hemi-parkinsonian mouse models with MPTP**

The hemi-parkinsonian mouse models were generated following the methods as described previously^3^. Briefly, 2 μl MPTP (21 μg μl^-1^ dissolved in normal saline) was injected into left striatum (AP: 0.4 mm, ML: 1.8 mm, DV: -3.5 mm) with a Hamilton syringe (5μl ,7633-01) controlled by a syringe pump (KD scientific, 788130) at a rate of 0.1 μl min^-1^. After injection, the syringe was left in place for 5 min to avoid backflow. The rotation numbers were calculated as ipsilateral rotations - contralateral rotations in 10 min after the injection of APO.

**Cannula implantation and drug delivery**

Mice were anaesthetized with 0.7% pentobarbital sodium (w/v) (70 mg/kg), and fixed on a stereotaxic frame (RWD Instruments, 68044). The skull was exposed and adjusted in parallel to the reference panel, and then a thin hole was made with a dental drill for injection. A single-guide cannula (RWD Instruments, 62001) was implanted 300 μm above the center of ANT according to the stereotaxic coordinate (AP: -0.7 mm, ML: 0.8 mm, DV: -3.5 mm), and fixed on the skull with dental cement and three anchoring skull screws (1mm diameter). After one week recovery, H-89 (10 μM, dissolved in aCSF, Tocris, 2910) or the cell-permeable peptide (10 μM) was pumped into ANT at the rate of 0.1 μl min^-1^ using syringe pump systems (RWD Instruments, R404). The cannula placement was checked by pumped aCSF diluted beads (Lumafluor, R180,1:100) into ANT after all experiments finished. Data were used only from animals with the correct placement.

**Brain nuclei lesion**

Lesions of the target nuclei were made by injecting ibotenic acid (IBO) following the methods as described previously^4^. Briefly, after anaesthetization, IBO (10 mg ml^-1^ and 0.5 μl, disolved in 0.2% ascorbic acid, Sigma, I2765) was injected into mouse STN (AP: -2.0 mm, ML:1.6 mm, DV: -4.5 mm) or ANT with a Hamilton syringe at a rate of 0.1 μl min^-1^. After injection, the syringe was left in place for 5 min.

**Virus injection**

After anaesthetized, the mice were fixed on a stereotactic frame. Viruses were injected into STN or ANT with a Hamilton syringe at a rate of 0.02 μl min^-1^. After injection, the syringe was left in place for 10 min to minimize the spread of viruses. The mice were used for behavioral tests or electrophysiological recording 3 weeks later. The following viruses were used in the current study; rAAV2/9-CaMKIIα-EYFP-WPRE-pA (STN, titre:1.19 × 10^13^ v.g.ml^-1^, 0.1μl; dilution:1:5 and 0.04μl for tissue clearing, BrainVTA PT-0102), rAAV2/1-CaMKIIα-Cre-WPRE-pA (STN, titre: 1.14 × 10^13^ v.g.ml^-1^, 0.1μl, BrainVTA PT-0220), rAAV-Ef1α-Dio-EYFP-WPRE-pA (ANT, titre: 5.35× 10^12^ v.g.ml^-1^, 0.1μl, BrainVTA PT-0012), rAAV/Retro-hSyn-Flpo-WPRE-pA (ANT, titre: 1.23× 10^13^ v.g.ml^-1^, 0.1μl, Taitool Bioscience S0271-2R-H20), rAAV-hSyn-Con/Fon-WPRE-pA (STN, titre: 1.14 × 10^13^ v.g.ml^-1^, 0.05μl, Taitool Bioscience S0267-9-H20), rAAV2/9-CaMKIIα-Cre-WPRE-pA (STN, titre:5.84×10^13^ v.g.ml^-1^,: 0.05μl, BrainVTA PT-0220), rAAV-CaMKIIα-hChR2(H134R)-mCherry-WPRE-pA (STN, titre:2×10^12^ v.g.ml^-1^, 0.1μl, BrainVTA PT-0279), rAAV-hSyn-eNpHR-YFP-WPRE-pA (STN or ANT, titre: 3.21×10^13^ v.g.ml^-1^, 0.1μl BrainVTA PT-0724), rAAV-Ef1α-Dio-eNpHR-EYFP-WPRE-pA (ANT, titre: 1×10^12^ v.g.ml^-1^, 0.1μl, BrainVTA PT-0006).

**Optogenetic surgery and light delivery**

The optogenetic manipulation was conducted following the methods as described previously^5^. Briefly, after anaesthetized, the mice were fixed on a stereotactic frame. An optical fiber with 200 μm in diameter (Newdoon Inc, ULC-589-200-0.73-4.0) was implanted 300 μm above the center of ANT of mice injected with different viruses. Finally, skull surface was covered by dental cement with three anchoring screws. Three weeks later, behavioral tests were performed. For mice expressing eNpHR and EYFP, 590 nm (continuous stimulation for eNpHR) LED light was delivered by wireless optical stimulation system (Newdoon Inc). Light intensity was calculated to be 5 mW.

**Immunofluorescence staining**

After anesthetized, mice were transcardially perfused with 0.9% NaCl followed by 4% (w/v) paraformaldehyde (PFA) in phosphate buffer solution (PBS, 0.01 M, for all experiments unless stated). Brains were dissected and fixed in 4% PFA for 6 hours at 4°C were then transferred into 20% (w/v) and 30% (w/v) sucrose in PBS for 36-48 hours. The brains were coronally sectioned at 40 μm in thickness with freezing microtome (Leica, CM1950) and the slices were collected for immunostaining. The slices were pre-incubated in 3% bovine serum albumin (w/v) with 0.5% Triton X-100 (v/v) for 2 hours at room temperature (RT), followed by overnight incubation at 4°C with relative primary antibodies (rabbit-anti-tyrosine hydroxylase antibody: Millipore, AB152, 1:1000; rabbit-anti-c-fos antibody: Santa Cruz, sc-52, 1:200; mouse-anti-NeuN antibody: Millipore, MAB377, 1:800; rabbit-anti-CaMKⅡ antibody: abcam, ab34703, 1:1000; rabbit-anti-GABA antibody: sigma, A2052, 1:1000; mouse-anti-Cre antibody: Millipore, MAB3120, 1:1000). Then, the slices were incubated with fluorescent secondary antibodies (Alexa fluor 488- or 633- conjugated goat anti-rabbit or anti-mouse (A11008, A11001, A21050); Alexa fluor 546- conjugated donkey anti-rabbit (A10040), from Invitrogen, all 1:2000) for 2 hours at RT. The primary antibodies and secondary antibodies were both diluted with PBS containing 3% bovine serum albumin (w/v) and 0.3% Triton X-100. The fluorescent signals were examined and photographed with an A1R laser-scanning confocal microscope (Nikon A1R, Japan). Unbiased cell quantification of positive neurons was counted manually.

**Western blot**

Briefly, the brain tissues containing ANT were homogenized in 1% sodium dodecyl sulfate (SDS). Twenty microliter protein aliquot from each sample were separated on 8% SDS-polyacrylamide gel electrophoresis and transferred to a polyvinylidene fluoride membrane. The membrane was blocked with 3% non-fat milk in PBS at RT for 2 hours and then incubated with the primary antibodies (rabbit-anti-AMPAR-GluR1, abcam, ab109450,1:1000; rabbit-anti-GluR1-S831, abcam, ab109464, 1:1000; rabbit-anti-GluR1-S845, abcam, ab76321, 1:1000) and mouse-anti-β-actin (Santa Cruz, sc47778,1:1000) overnight at 4°C. After washing three times, the membrane was incubated with HRP at RT, and the protein bands were then visualized by the ECL system (Tanon Inc, 5200) and band gray scale were quantified with ImageJ software.

**Behavioral assays**

**Balance beam test** The balance beam test was conducted following the methods as described previously^6,7^ with a modification. Briefly, the apparatus consists of a 1 m round beam (12mm in diameter) resting 50 cm above the ground. A black box containing nesting material was placed at the end of the beam as the destination. A lamp, as an aversive stimulus, was placed at the origin side. Before testing, mice were trained 3 days to accommodate. For training, each mouse passed through the beam three times every day with an interval of 10 min. After three days training, the test was performed and the time passing through the beam was recorded as the measure of mobility of each mouse.

**Apomorphine-induced rotation test** The rotation test, a paradigm routinely used both to evaluate the establishment of hemi-parkinsonian model and effects, was conducted following the methods as described previously^2,8^. Briefly, mice were injected with apomorphine (0.5 mg kg^-1^, intraperitoneally) and put in a cylinder of 60 cm in diameter. Rotation numbers were counted manually 3 min after apomorphine injection. The average number per minute was calculated.

**In vivo electrophysiological recording**

**Electrode implantation** Two bundles of recording electrodes were implanted into bilateral ANT according to the stereotaxis coordinate (AP: -1.65 ~ -1.72 mm, ML: ±1.25 ~ 1.6 mm, DV: -5.2 ~ -5.4 mm). Each bundle contained 7 to 13 Teflon-insulated tungsten microwires and was threaded through a 26-gauge stainless steel guide tube with longer than 2 mm tips left outside. Two copper wires were connected to the skull screw and severed as ground and reference.

**Electrophysiological recording***.* Two weeks after recovery from the electrode implantation, in vivo recording was performed during which rats were able to freely move in a cylinder with 60 cm diameter. At least 3 recording sessions with 10 min for each were conducted. The behavior of rats was monitored and recorded. The spike data was sampled by 30 kS/s with 250-5k Hz bandpass filter. The local filed potential (LFP) data was sampled by 2 kS/s with 250 Hz lowpass filter (CerePlex Direct, Blackrock Microsystems, USA).

**Spike analysis** Single units were off-line sorted by wavelets and superparamagnetic clustering method^9^. For good unit isolation, units with more than 5% spikes with less than 3 ms refractory period in autocorrelation histogram were excluded. In total, we got 13 single units from 11 PD modeled rats.

**LFP analysis** The LFP data were firstly filtered by a 1 Hz highpass filter and 50 Hz bandstop filter to rule out the very low frequency components and 50 Hz electric noise. The power of LFP signal was calculated by Fast Fourier Transformation (FFT). An increase of power was found in high-beta band in the ipsilateral ANT of PD model rats. Thus, the total power between 15 Hz to 35 Hz was compared between PD model and normal rats.

**Verification of electrode site** After recording, anodal electrolytic lesion of the recording sites was done by injecting 30~50 μA current for 30 s. Then, the rats were transcardially perfused with 0.9% NaCl and 4% PFA sequentially. The brain was post-fixed in 4% PFA for 12 hours and then transferred into 20% sucrose for 2 days. The brain was frozen sectioned into 40 μm slices and processed with CaMK II immunostaining. The penetration tracks and lesion sites were observed and photographed by laser-scanning microscope and digital camera.

**In vitro** **electrophysiological recording**

**Slice preparation** Electrophysiological recordings were conducted following the methods as described previously^10,11^. Briefly, after anaesthetized, the mouse brain was dissected out as quickly as possible. Coronal slices containing the ANT (300 μm) were obtained using the vibratome (Leica VT1000S, Germany) in oxygenated ice-cold sucrose-based dissection solution ((in mM): 213 sucrose, 2.5 KCl, 2 MgSO_4_, 2 CaCl_2_, 1.25 NaH_2_PO_4_, 26 NaHCO_3_ and 10 glucose). Slices were incubated and recovered at 34°C for about 40 min and kept at RT until transfer to the recording chamber in artificial cerebrospinal fluid (aCSF) containing (in mM):126 NaCl, 2.5 KCl, 2 MgSO_4_, 2 CaCl_2_, 1.25 NaH_2_PO_4_, 26 NaHCO_3_ and 25 glucose. Recordings were performed with aCSF at a flow rate of 1.5-2 ml min^-1^. All solutions were continuously bubbled with 95% O_2_ + 5% CO_2_ during the experiments. Osmotic pressure was 290 - 310 mOsm.

**In vitro recording** To examine the functional connection between STN and ANT, we recorded the evoked EPSC of ANT neurons in a voltage-clamp mode at a holding potential of -70 mV while activating ChR2 on the mCherry-positive fibers projected from STN to ANT with blue light (470 nm, 0.1 Hz, 2ms). To further verify the excitatory connection between STN and ANT, the CNQX (10 μM, abcam, 120044) was added into aCSF to block AMPAR. To further verify the functional connection, TTX (1 μM) and 4-AP (100 μM, sigma, 275875) was added into aCSF. Recording pipettes (3.5-5 MΩ) were filled with intracellular solution containing (in mM): 140 potassium gluconate, 3 KCl, 2 MgCl_2_, 10 HEPES, 0.2 EGTA, 2 Na_2_ATP (pH 7.3). To examine the validity of NpHR, 100-200 pA current was injected to induce action potentials and observed change of action potentials when stimulation with 590 nm yellow light. Evoked AMPAR and NMDAR EPSCs were recorded under the potential of -70 mV and 40 mV, respectively. A tungsten bipolar stimulating electrode (FHC, Bowdoin, ME) or optical fiber was placed in ANT, 10~50mm laterally to the cell body. The EPSC was pharmacologically isolated by blocking GABA_A_ receptors with picrotoxin (100 μM, Sigma, p1675). The ratio of AMPAR/NMDAR was calculated by dividing the peak of the AMPAR-mediated EPSC at −70 mV by the value of the NMDAR-mediated EPSC after stimulation start time 50ms at +40 mV. Rectification index was measured by the ratio of AMPAR mediated-EPSC recorded at -70 mV and +40 mV in the presence of D-APV (50 μM) (abcam, ab120003) and picrotoxin. Recording pipettes (3.5-5MΩ) were filled with intracellular solution containing (in mM): 122 CsMeSO3, 3.7 NaCl, 20 HEPES, 10 BAPTA, 0.2 EGTA, 0.3 MgATP, 0.3 Na_3_GTP, 5 TEA-Cl, 0.1 spermine, 5 Qx-314-Br. The neuronal leak currents for analysis were <100pA. Data were acquired using HEKA EPC10 (HEKA, Germany), sampled at 10 kHz, and filtered at 2 kHz. Off-line analysis was done by using PATCHMASTER (HEKA, Germany). The cell series resistance was <30 MΩ. The data that showed >20% change of series resistance was excluded from analysis.

**Statistical analysis**

Unpaired and paired two-sample Student’s t-test were used for comparison within two groups. Two-way analysis of variance (ANOVA) with Bonferroni post hoc analysis were used for multiple group comparison. Mann-Whitney test was used for analysis of firing rates from in vivo recording. All the analyses were performed by using Graphpad Prism and all data were presented as the mean ± SEM. expect firing rates from in vivo recording which were presented as median (interquartile range).

**Figure. S1.**


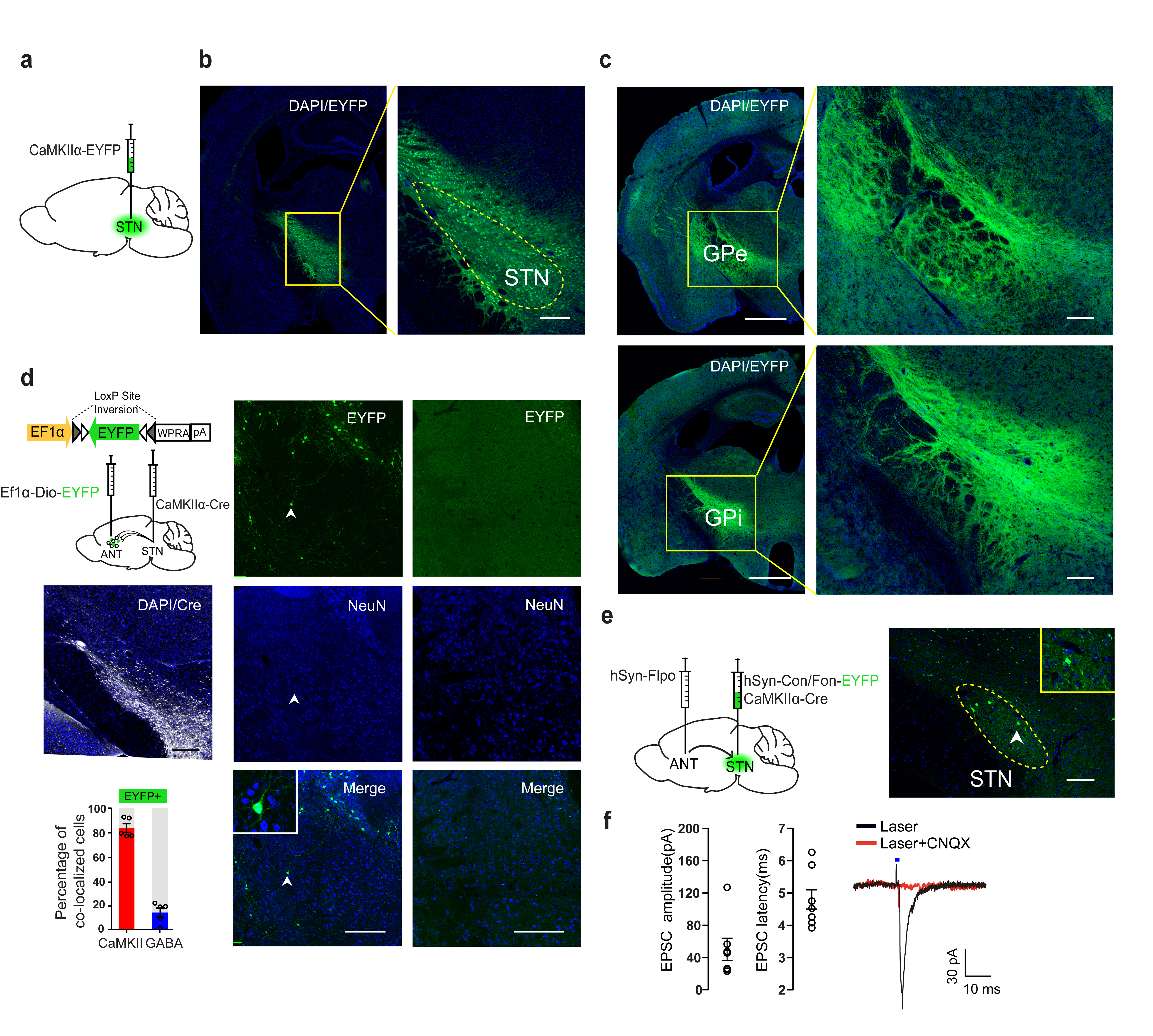


**Figure. S1. A newly identified projection of STN to ANT. a.** Schematics of AAV-CaMKIIα-EYFP injection into mouse unilateral STN. **b.** EYFP expression in STN in coronal brain slices. *n* = 5 mice. Scale bar, 200 μm. **c.** Representative images of EYFP-positive fibers observed in GPe, GPi and their magnified views (middle). *n* = 5 mice. Scale bar, 1000 and 200μm, respectively. **d.** Schematics of dual virus injections (top, left), the representative image of STN injected with trans-monosynaptic AAV expressing Cre recombinase (middle, left) and statistics of the percentage of EYFP-positive cells co-localized with CaMKII (83.68% ± 3.518%) or GABA (14.12% ± 3.770%) (bottom, left). Each circle represents a mouse. Data are mean ± SEM of 5 mice. Representative images of EYFP-positive ANT cells stained with anti-NeuN antibody. **Inset:** magnified views of arrow head regions. Scale bar, 200 μm (middle). Control: virus expressing Dio-EYFP injected into ANT, Scale bar, 200 μm (right). **e.** Schematics of multi virus injections (left). The representative image of EYFP-positive cells in STN (right). **Inset**: magnified views of arrow head regions. *n* = 5 mice. Scale bar, 100 μm. **f.** Statistics of amplitude and latency of the evoked EPSCs by optical stimulation in ANT slices (Blue bar: 470 nm, 2 ms) (left). Each circle represents a neuron. Data are mean ± SEM of 7 neurons from 3 mice. Representative trace of the evoked EPSC in the ANT neuron in the presence of CNQX (10 μM) (right).

**Figure. S2.**


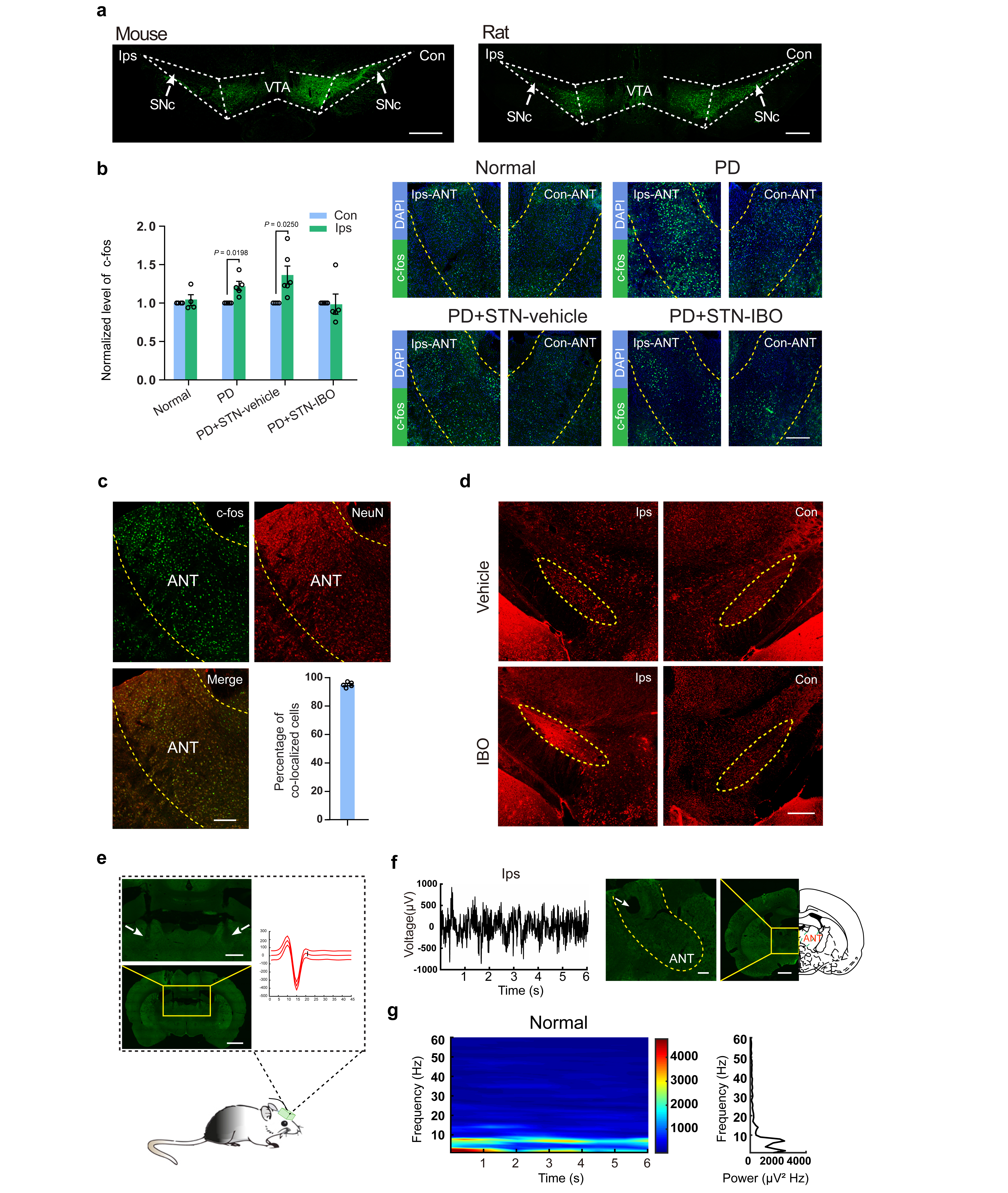


**Figure. S2. Activity of ipsilateral ANT is increased in PD model rodents. a.** Representative images of SNc immunostained with anti-tyrosine hydroxylase antibody in the mouse (left) and rat (right) after injection of 6-OHDA. Scale bar, 500 μm. **b.** Statistics of the percentage of c-fos-positive cells in ipsilateral (Ips) ANT relative to that in contralateral (Con) ANT and normalized to Con (left). Images of co-staining of c-fos and DAPI of ipsilateral and contralateral ANT in normal, PD model mice (top, right) or PD model mice with STN injected with vehicle or IBO (bottom, right). Each circle represents a mouse. *n* = 4-6 mice for each group. Scale bar, 200 μm. **c.** Representative images of ipsilateral ANT slices from PD model mice stained with the antibodies against c-fos (left, top), NeuN (right, top) and merged images (left, bottom). Scale bar, 100 μm. Statistics of the percentage of c-fos expressing cells co-localized with NeuN (right, bottom). Each circle represents a mouse. Data are mean ± SEM of at least three independent experiments with indicated 5 mice. **d.** Representative images of STN slices obtained from the mice injected with vehicle (top) or IBO (bottom) to ipsilateral STN and stained with anti-NeuN antibody. The white arrow points to the lesion area. Scale bar, 100 μm. *n* = 5 mice. **e.** Demonstration of *in vivo* multi-channel electrophysiological recordings in freely moving rats. Left: electrodes implanted in bilateral ANTs, and the white arrow points to the location of recording site with a lesion made (arrow) after finishing recording. Scale bar, 200 μm (top) and 500 μm (bottom); Right: representative spike waveform of one neuron recorded from ANT. **f.** Representative raw trace of LFP following time (top) and slice of a rat with electrodes implanted in the ipsilateral ANT (bottom). The white arrow points to the location of the recording site with a lesion made (arrow) after finishing recording. Scale bar, 100 μm (left, bottom) and 500 (right, bottom) μm respectively. **g.** Spectrogram and power spectrum density analysis of local field potentials in normal rats.

**Figure. S3.**


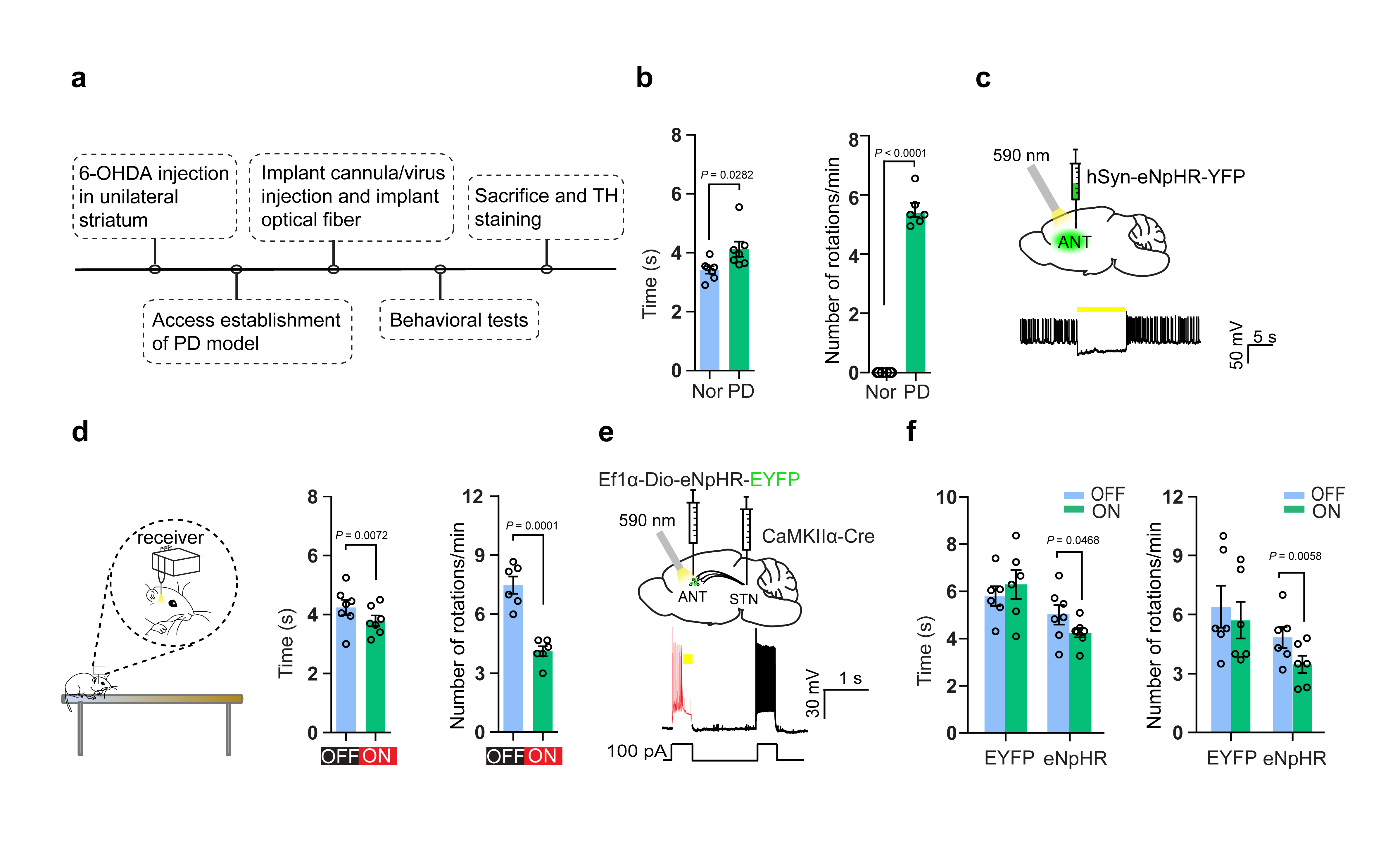


**Figure. S3. Inhibiting STN-ANT activities corrected the motor behaviors in PD models. a.** Schematics of the experiments. **b.** Time of passing the beam and the number of APO-induced rotations in the mice. **c.** Schematics of wireless optogenetic manipulation on the activity of ipsilateral ANT injected with the virus expressing eNpHR (top) and functional verification of eNpHR (yellow bar: 590 nm, continuous) (bottom). *n* = 3 mice. **d.** Time of passing the beam and the number of APO-induced rotations by wireless optogenetic manipulation on ipsilateral ANT activity in PD model mice. **e.** Schematics of optogenetic manipulation on STN-projected ANT neuron activities (top) and functional verification of eNpHR (yellow bar: 590 nm, continuous) (bottom). *n* = 3 mice. **f.** Time of passing the beam and the number of APO-induced rotations of PD model mice. Each circle in all panels represents a mouse. Data are mean ± SEM of at least three independent experiments.

**Figure. S4.**


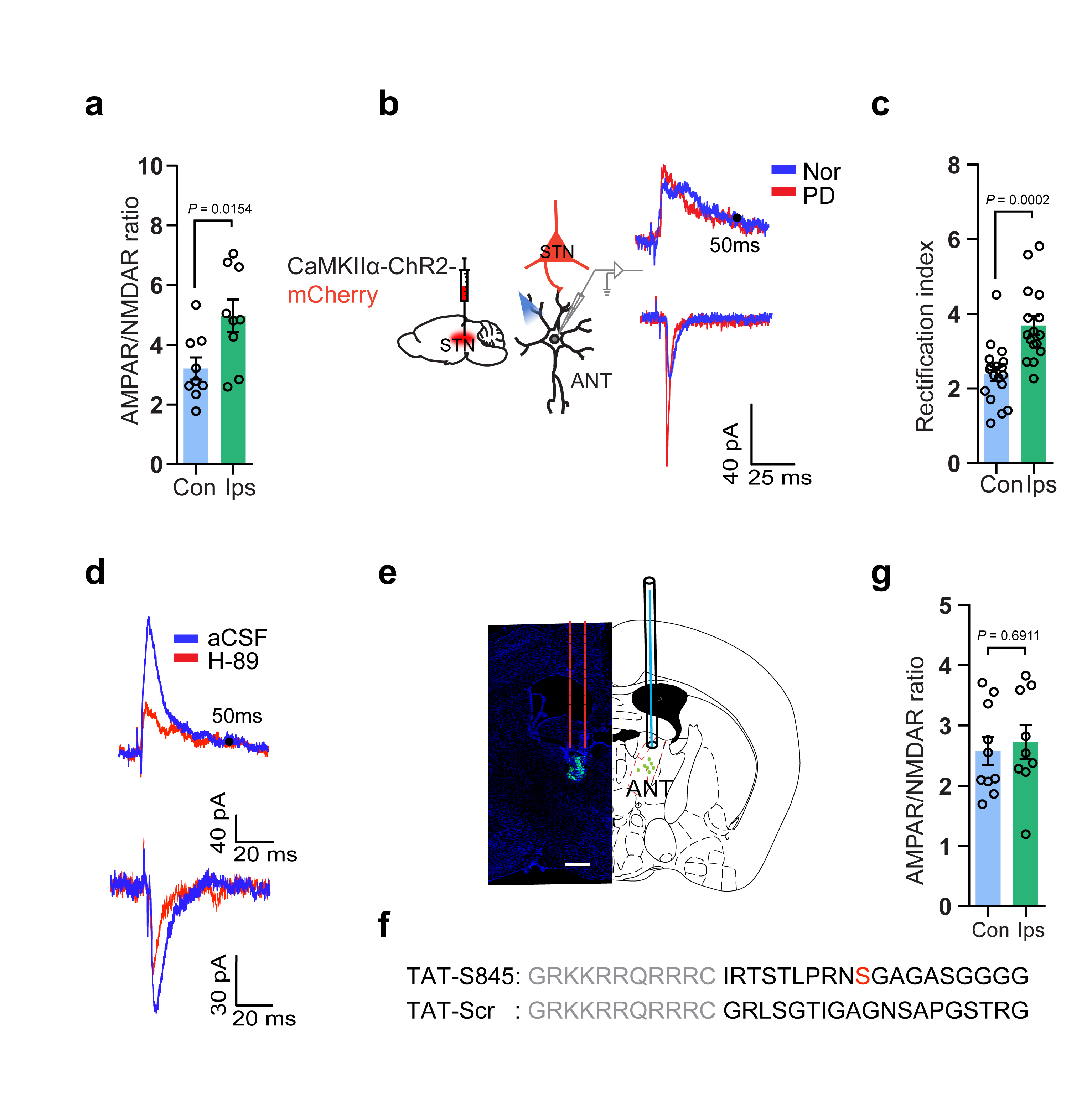


**Figure. S4. STN-ANT synaptic plasticity is crucial for PD motor deficits a.** Ratio of AMPAR/NMDAR currents evoked by electrical stimulation in contralateral and ipsilateral ANT neurons of the PD mice. *n* = 4-6 mice in each group. **b.** schematics of optical stimulation of STN projection fibers combined with whole cell recording on ANT slices (left), representative trace of evoked AMPAR currents holding at -70 mV and evoked NMDAR currents holding at +40 mV of normal mice (blue) or PD mice (red)(right). **c.** Rectification index evoked by electrical stimulation in contralateral and ipsilateral ANT neurons of the PD mice. *n* = 4-6 mice in each group. **d.** Representative trace of evoked AMPAR currents by electrical stimulation holding at -70 mV and evoked NMDAR currents holding at +40 mV in the presence of aCSF (blue) or H-89 (red) (right). **e.** Representative infusion site verified by fluorescent beads injected (1 μl) into ipsilateral ANT. Scale bar, 500 μm. **f.** The sequence of TAT-S845 and its scramble (scr). **g.** The ratio of AMPAR/NMDAR current evoked by electrical stimulation in contralateral and ipsilateral GPi neurons. *n* = 7 mice. Each circle in all panels represents a neuron. Data are mean ± SEM.

**References**

1 Tieu, K. A guide to neurotoxic animal models of Parkinson's disease. *Cold Spring Harb Perspect Med* **1**, a009316, doi:10.1101/cshperspect.a009316 (2011).

2 Iancu, R., Mohapel, P., Brundin, P. & Paul, G. Behavioral characterization of a unilateral 6-OHDA-lesion model of Parkinson's disease in mice. *Behav Brain Res* **162**, 1-10, doi:10.1016/j.bbr.2005.02.023 (2005).

3 Fung, Y. K. & Troxel, C. E. Amphetamine-induced circling behaviour in MPTP-lesioned mice. *J Pharm Pharmacol* **37**, 922-923, doi:10.1111/j.2042-7158.1985.tb05005.x (1985).

4 Barthas, F. *et al.* The anterior cingulate cortex is a critical hub for pain-induced depression. *Biol Psychiatry* **77**, 236-245, doi:10.1016/j.biopsych.2014.08.004 (2015).

5 Felix-Ortiz, A. C. *et al.* BLA to vHPC inputs modulate anxiety-related behaviors. *Neuron* **79**, 658-664, doi:10.1016/j.neuron.2013.06.016 (2013).

6 Luong, T. N., Carlisle, H. J., Southwell, A. & Patterson, P. H. Assessment of motor balance and coordination in mice using the balance beam. *J Vis Exp*, doi:10.3791/2376 (2011).

7 Allbutt, H. N. & Henderson, J. M. Use of the narrow beam test in the rat, 6-hydroxydopamine model of Parkinson's disease. *J Neurosci Methods* **159**, 195-202, doi:10.1016/j.jneumeth.2006.07.006 (2007).

8 Kelly, P. H. Unilateral 6-hydroxydopamine lesions of nigrostriatal or mesolimbic dopamine-containing terminals and the drug-induced rotation of rats. *Brain Res* **100**, 163-169, doi:10.1016/0006-8993(75)90253-x (1975).

9 Quiroga, R. Q., Nadasdy, Z. & Ben-Shaul, Y. Unsupervised spike detection and sorting with wavelets and superparamagnetic clustering. *Neural Comput* **16**, 1661-1687, doi:10.1162/089976604774201631 (2004).

10 Feng, S. *et al.* Sonic hedgehog is a regulator of extracellular glutamate levels and epilepsy. *EMBO Rep* **17**, 682-694, doi:10.15252/embr.201541569 (2016).

11 Yin, L. *et al.* Autapses enhance bursting and coincidence detection in neocortical pyramidal cells. *Nat Commun* **9**, 4890, doi:10.1038/s41467-018-07317-4 (2018).
