# Supplementary figures and images for: STN–ANT plasticity is crucial for the motor control in Parkinson’s disease model
Source: Signal Transduct Target Ther. 2021 Jun 9;6:215. doi: 10.1038/s41392-021-00545-z (PMC8187716; doi:10.1038/s41392-021-00545-z)

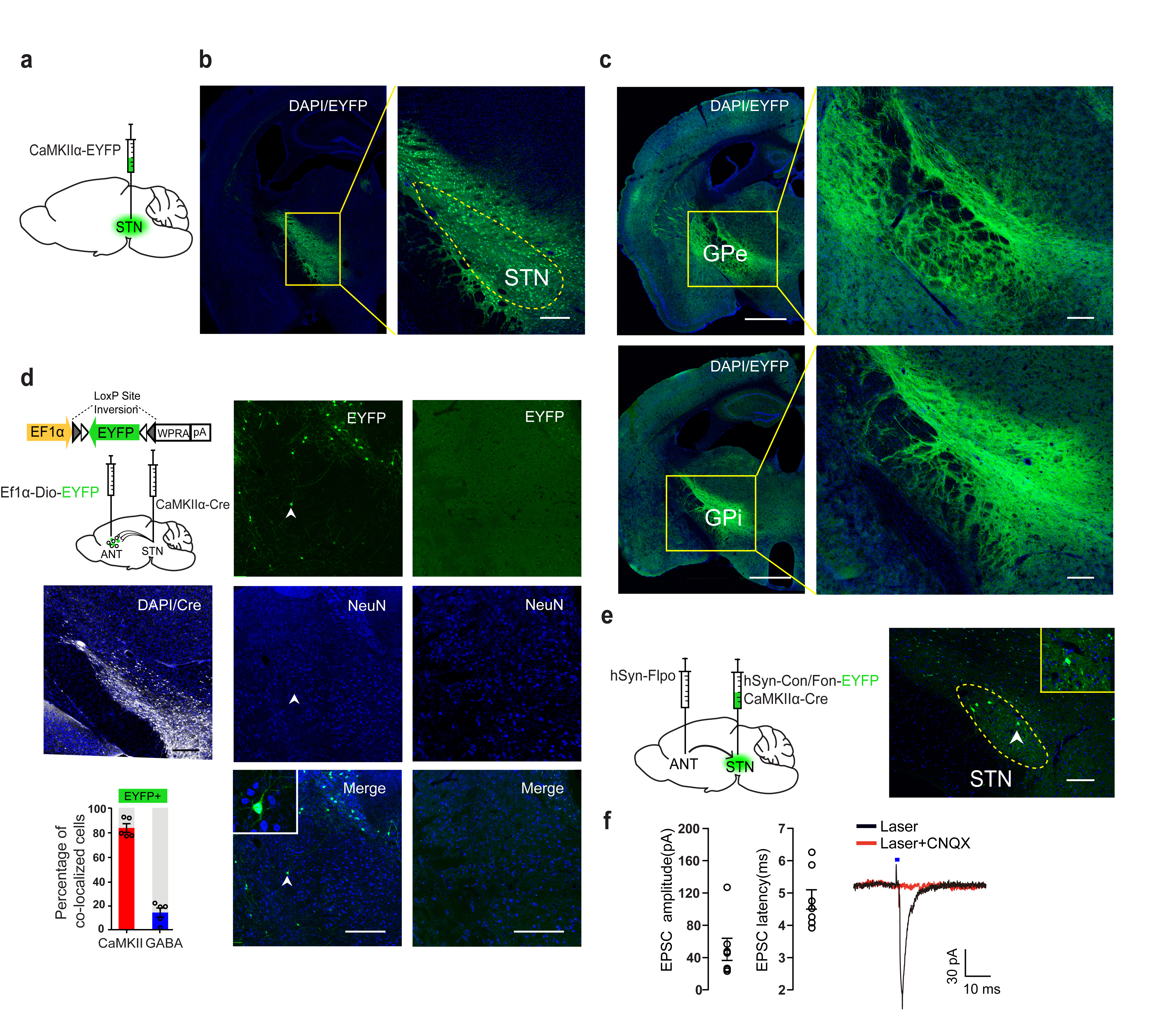

Supplement: Supplementary file 3 — supplementary Figure1a-f [file 41392_2021_545_MOESM3_ESM.jpg]

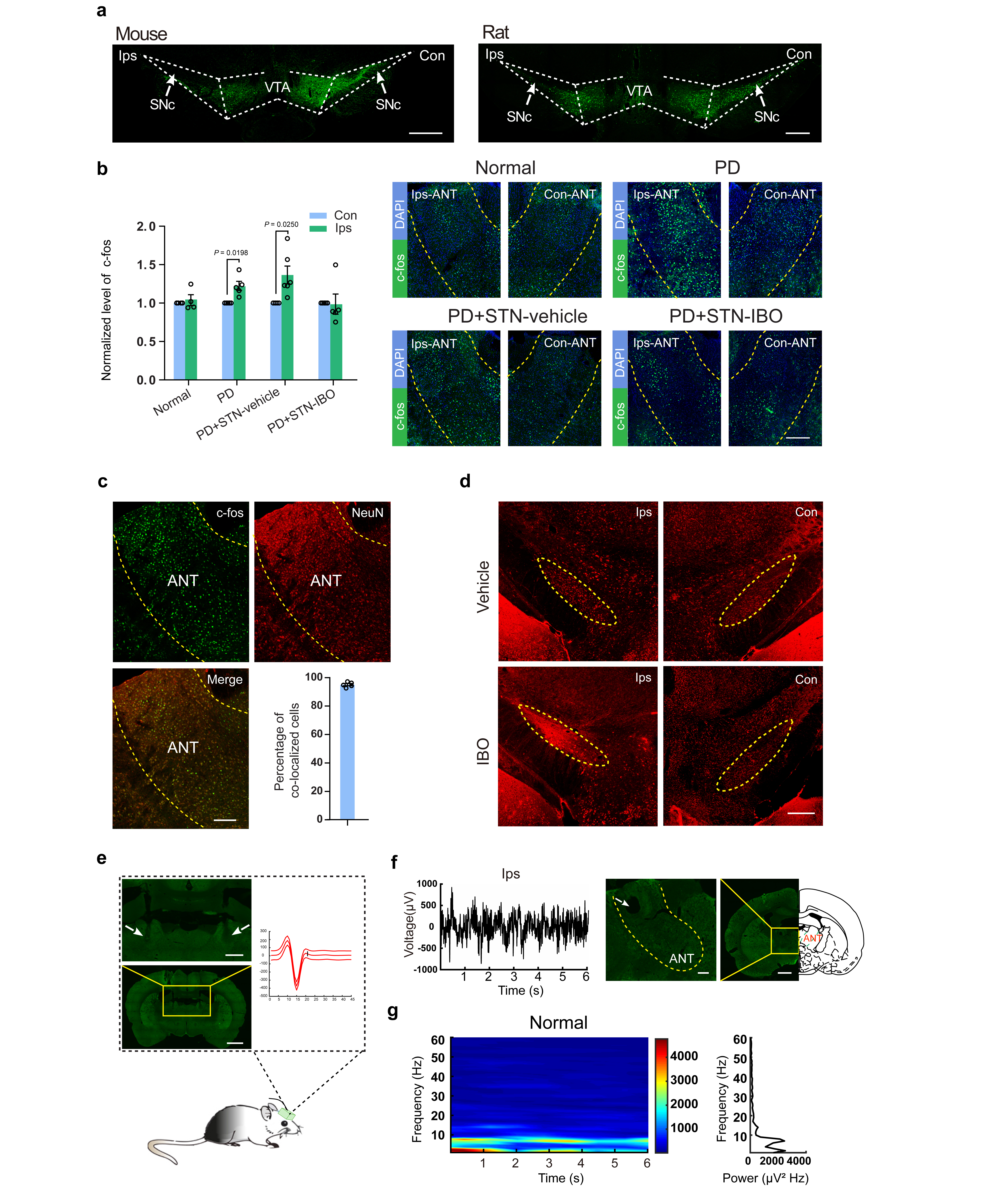

Supplement: Supplementary file 4 — supplementary Figure2a-g [file 41392_2021_545_MOESM4_ESM.jpg]

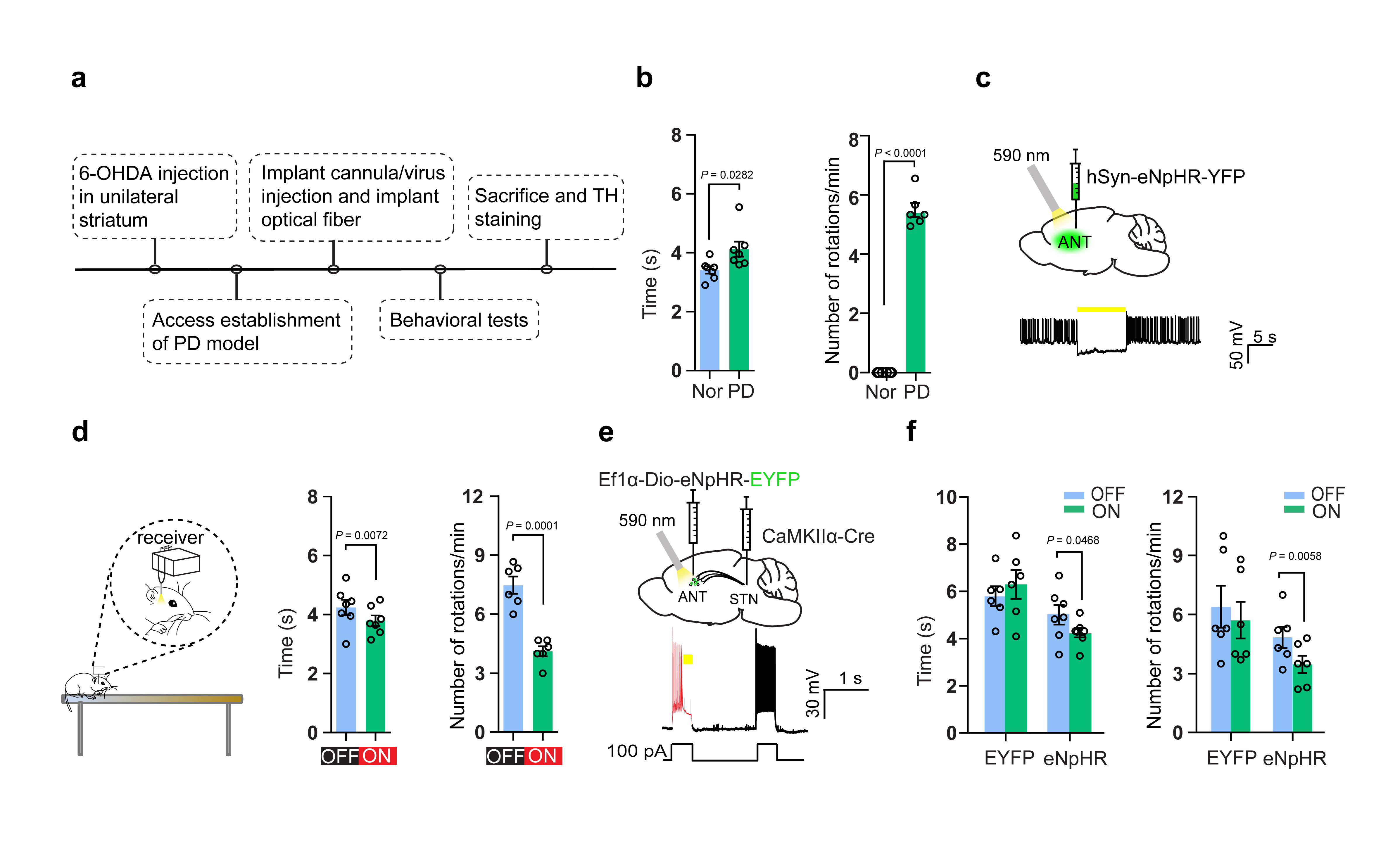

Supplement: Supplementary file 5 — supplementary Figure3a-f [file 41392_2021_545_MOESM5_ESM.jpg]

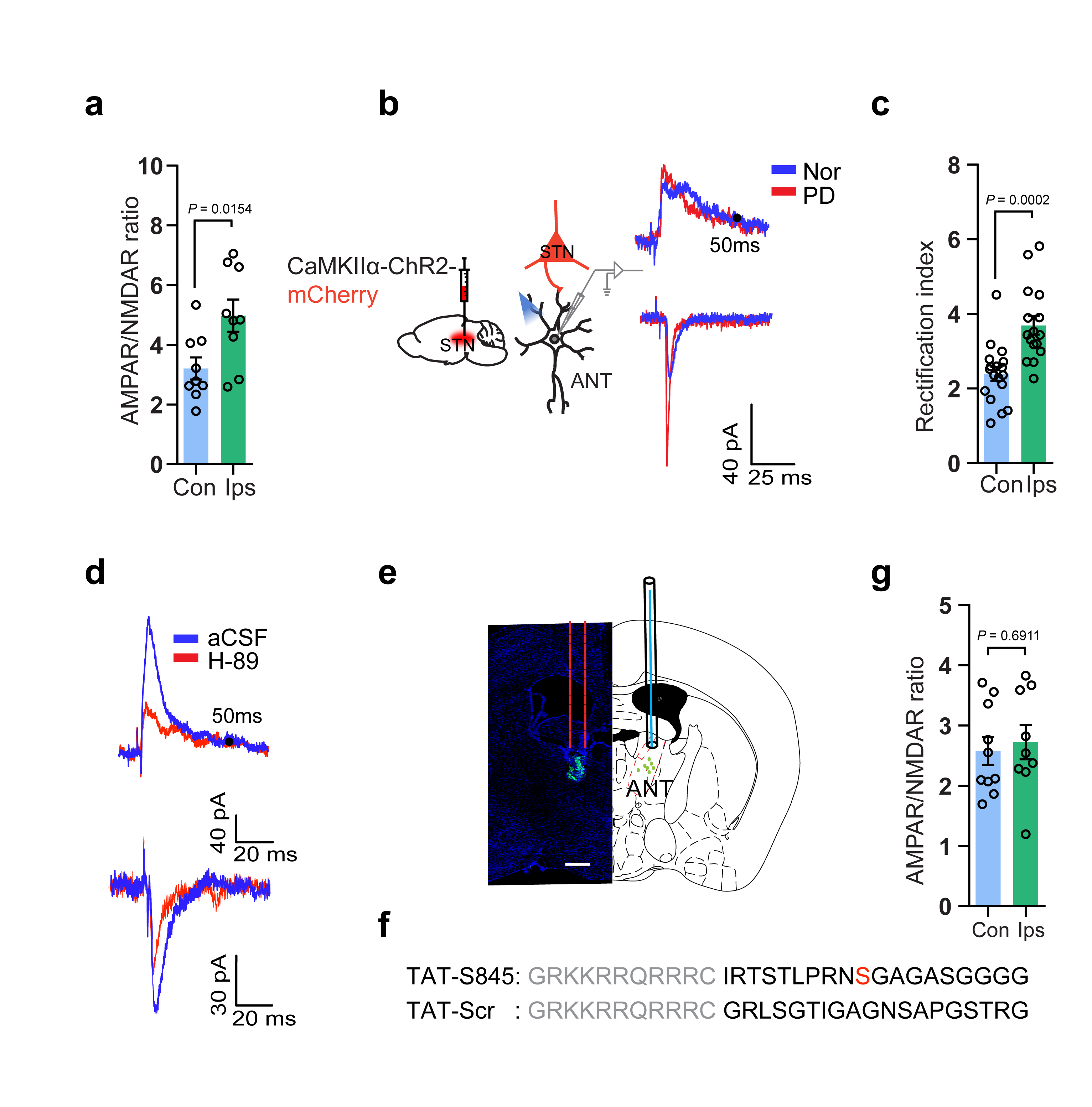

Supplement: Supplementary file 6 — supplementary Figure4a-g [file 41392_2021_545_MOESM6_ESM.jpg]
