# Supplementary material for: STN–ANT plasticity is crucial for the motor control in Parkinson’s disease model
Source: Signal Transduct Target Ther. 2021 Jun 9;6:215. doi: 10.1038/s41392-021-00545-z (PMC8187716; doi:10.1038/s41392-021-00545-z)
Supplement: Supplementary file 7 — Supplementary information [file 41392_2021_545_MOESM7_ESM.docx]

**Supplementary information**


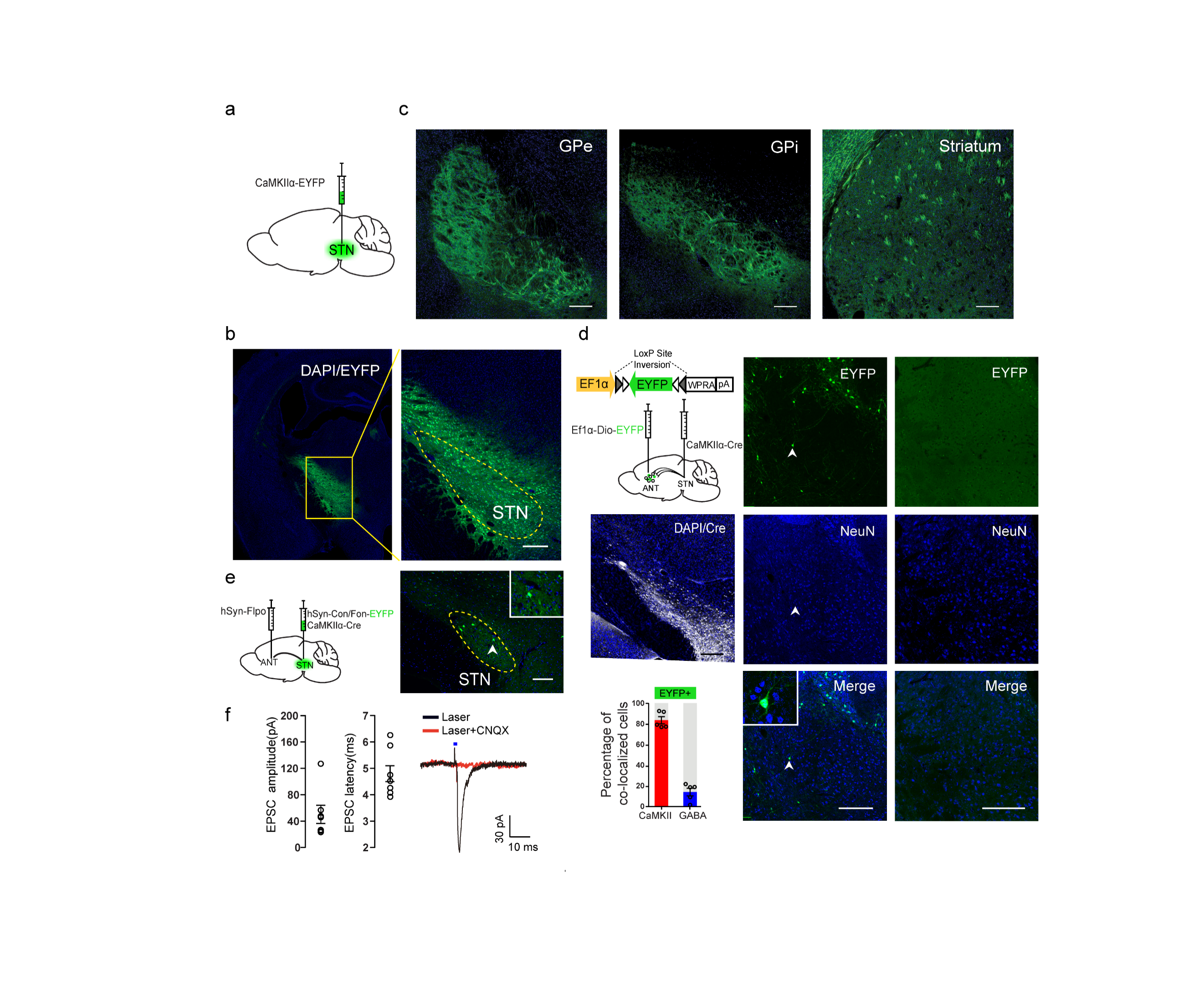


**Supplemental Fig 1 (Fig. S1). A newly identified projection of STN to ANT.** a. Schematics of AAV-CaMKIIα-EYFP injection into mouse unilateral STN. b. EYFP expression in STN in coronal brain slices. *n* = 5 mice. Scale bar, 200 μm. c. Representative images of EYFP-positive fibers observed in GPe, GPi and striatum. *n* = 5 mice. Scale bar, 100 μm. d. Schematics of dual virus injections (top, left), the representative image of STN injected with trans-monosynaptic AAV expressing Cre recombinase (middle, left) and statistics of the percentage of EYFP-positive cells co-localized with CaMKII (83.68% ± 3.518%) or GABA (14.12% ± 3.770%) (bottom, left). Each circle represents a mouse. Data are mean ± SEM of 5 mice. Representative images of EYFP-positive ANT cells stained with anti-NeuN antibody. Inset: magnified views of arrow head regions. Scale bar, 200 μm (middle). Control: virus expressing Dio-EYFP injected into ANT, Scale bar, 200 μm (right). e. Schematics of multi virus injections (left). The representative image of EYFP-positive cells in STN (right). Inset: magnified views of arrow head regions. *n* = 5 mice. Scale bar, 100 μm. f. Statistics of amplitude and latency of the evoked EPSCs by optical stimulation in ANT slices (Blue bar: 470 nm, 2 ms) (left). Each circle represents a neuron. Data are mean ± SEM of 7 neurons from 3 mice. Representative trace of the evoked EPSC in the ANT neuron in the presence of CNQX (10 μM) (right).


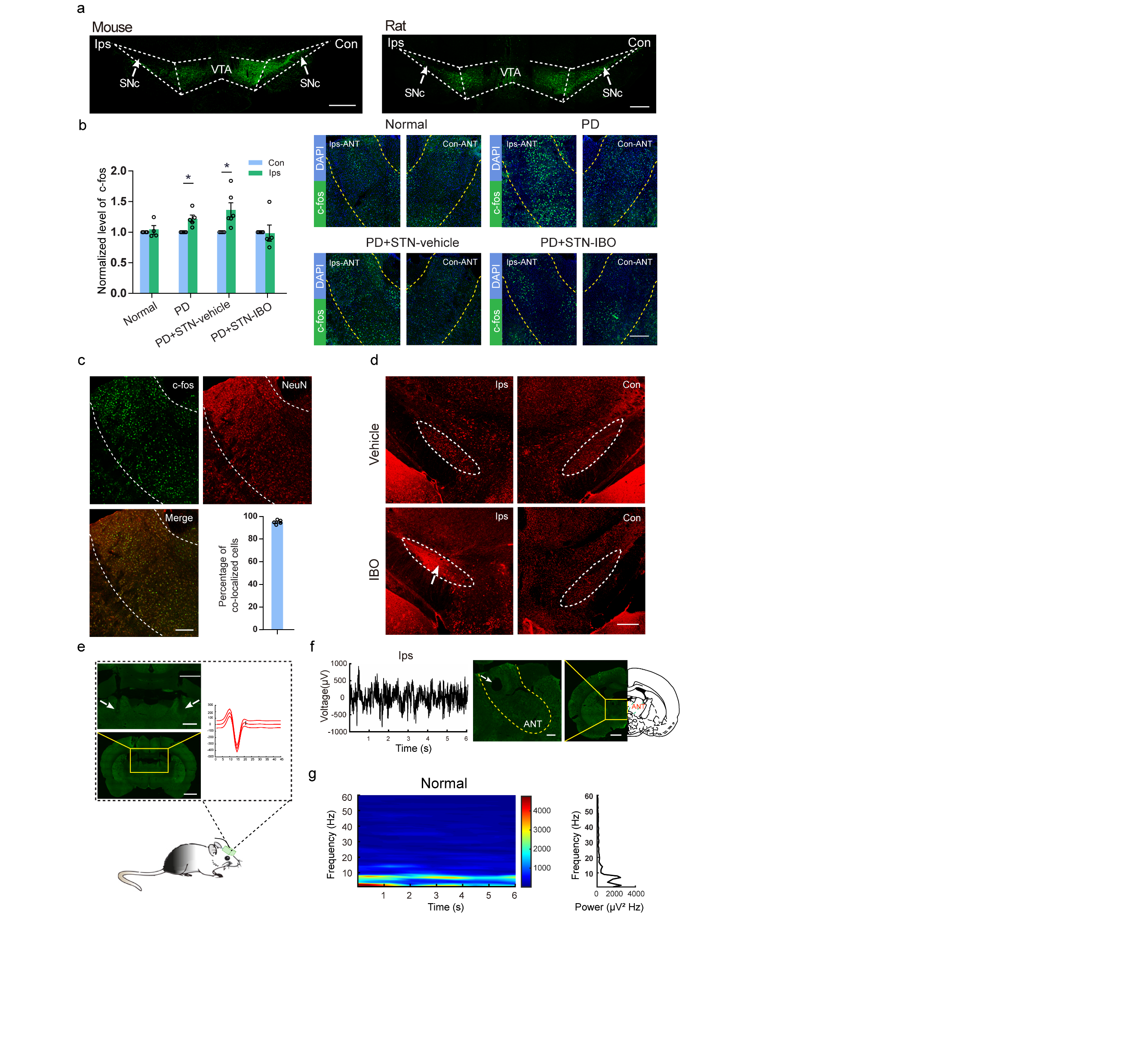


**Supplemental Fig 2 (Fig. S2). Activity of ipsilateral ANT is increased in PD model rodents.** a. Representative images of SNc immunostained with anti-tyrosine hydroxylase antibody in the mouse (left) and rat (right) after injection of 6-OHDA. Scale bar, 500 μm. b. Statistics of the percentage of c-fos-positive cells in ipsilateral (Ips) ANT relative to that in contralateral (Con) ANT and normalized to Con (left). Images of co-staining of c-fos and DAPI of ipsilateral and contralateral ANT in normal, PD model mice (top, right) or PD model mice with STN injected with vehicle or IBO (bottom, right). Each circle represents a mouse. *n* = 4-6 mice for each group. Scale bar, 200 μm. c. Representative images of ipsilateral ANT slices from PD model mice stained with the antibodies against c-fos (left, top), NeuN (right, top) and merged images (left, bottom). Scale bar, 100 μm. Statistics of the percentage of c-fos expressing cells co-localized with NeuN (right, bottom). Each circle represents a mouse. Data are mean ± SEM of at least three independent experiments with indicated 5 mice. d. Representative images of STN slices obtained from the mice injected with vehicle (top) or IBO (bottom) to ipsilateral STN and stained with anti-NeuN antibody. The white arrow points to the lesion area. Scale bar, 100 μm. *n* = 5 mice. e. Demonstration of *in vivo* multi-channel electrophysiological recordings in freely moving rats. Left: electrodes implanted in bilateral ANTs, and the white arrow points to the location of recording site with a lesion made (arrow) after finishing recording. Scale bar, 200 μm (top) and 500 μm (bottom); Right: representative spike waveform of one neuron recorded from ANT. f. Representative raw trace of LFP following time (top) and slice of a rat with electrodes implanted in the ipsilateral ANT (bottom). The white arrow points to the location of the recording site with a lesion made (arrow) after finishing recording. Scale bar, 100 μm (left, bottom) and 500 (right, bottom) μm respectively. g. Spectrogram and power spectrum density analysis of local field potentials in normal rats.


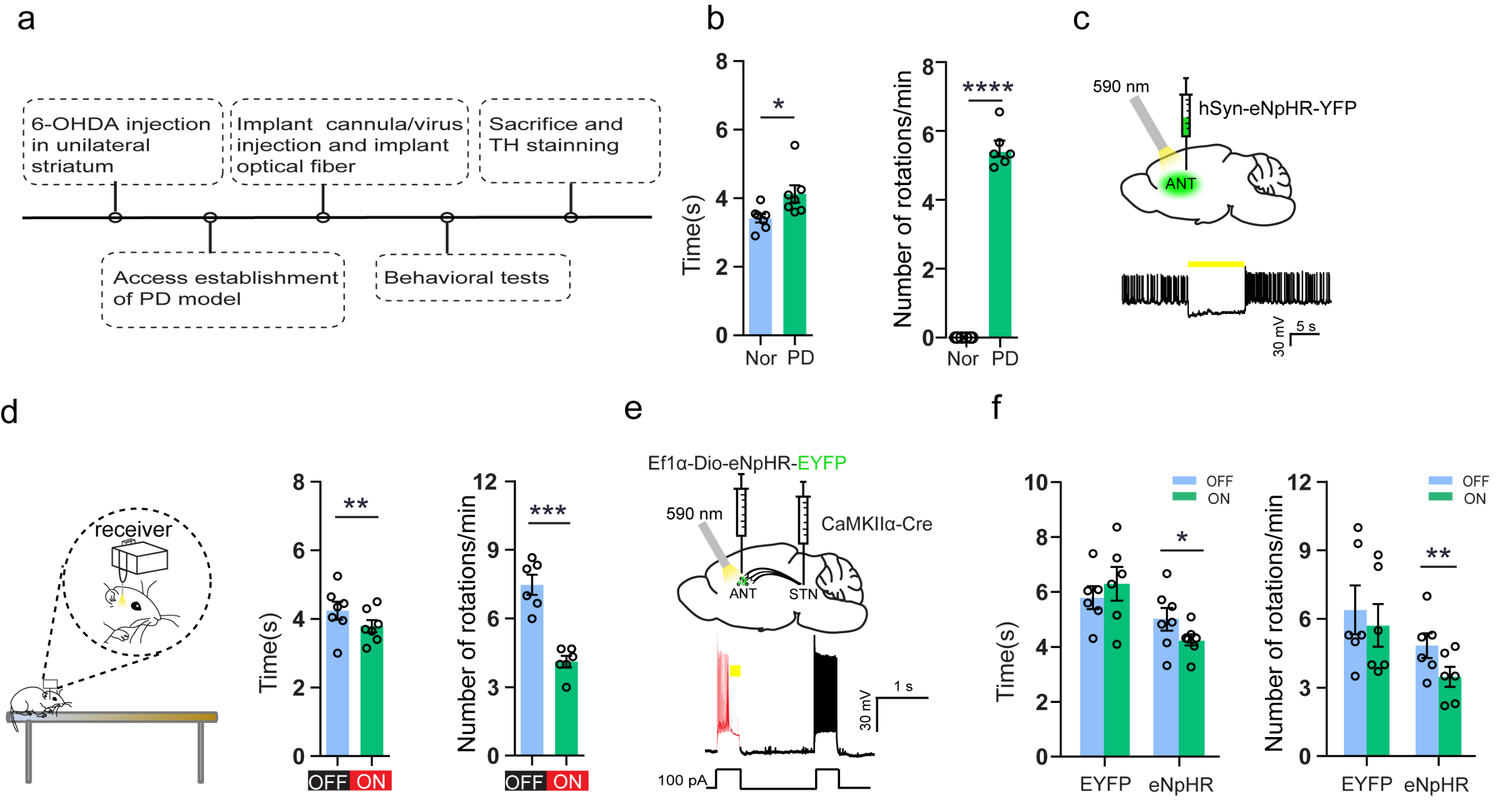


**Supplemental Fig 3 (Fig. S3). Inhibiting STN-ANT activities corrected the motor behaviors in PD models.** a. Schematics of the experiments. b. Time of passing the beam (Left: Normal (Nor): 3.41 ± 0.12 s, *n* = 7; PD: 4.12 ± 0.25 s, *n* = 7, *P* = 0.028) and the number of APO-induced rotations in the mice (Right: Nor: 0.0 ± 0.0, *n* = 6; PD: 5.49 ± 0.23; *n* = 6; *P* < 0.0001). c. Schematics of wireless optogenetic manipulation on the activity of ipsilateral ANT injected with the virus expressing eNpHR (top) and functional verification of eNpHR (yellow bar: 590 nm, continuous) (bottom). *n* = 3 mice. d. Time of passing the beam (Left: laser off: 4.24 ± 0.26 s; laser on: 3.79 ± 0.18 s; *n* = 7, *P =* 0.0072) and the number of APO-induced rotations (Right, laser off: 7.47 ± 0.44; laser on: 4.11 ± 0.25; *n* = 6, *P* = 0.0001) by wireless optogenetic manipulation on ipsilateral ANT activity in PD model mice. e. Schematics of optogenetic manipulation on STN-projected ANT neuron activities (top) and functional verification of eNpHR (yellow bar: 590 nm, continuous) (bottom). *n* = 3 mice. f. Time of passing the beam (EYFP: laser off: 5.79 ± 0.42 s; laser on: 6.30 ± 0.62 s; *n* = 6, *P* = 0.266. eNpHR: laser off: 5.00 ± 0.41 s; laser on: 4.25 ± 0.21 s, *n* = 7, *P* =0.0418) and the number of APO-induced rotations (EYFP: laser off: 6.40 ± 1.07; laser on: 5.72 ± 0.93, *n* = 6, *P* = 0.0607. eNpHR: laser off: 4.83 ± 0.53; laser on: 3.46 ± 0.44; *n* = 6, *P* = 0.0058) of PD model mice. Each circle in all panels represents a mouse. Data are mean ± SEM of at least three independent experiments. **P* < 0.05; ** *P* < 0.01; ****P* < 0.001; *****P* < 0.0001.


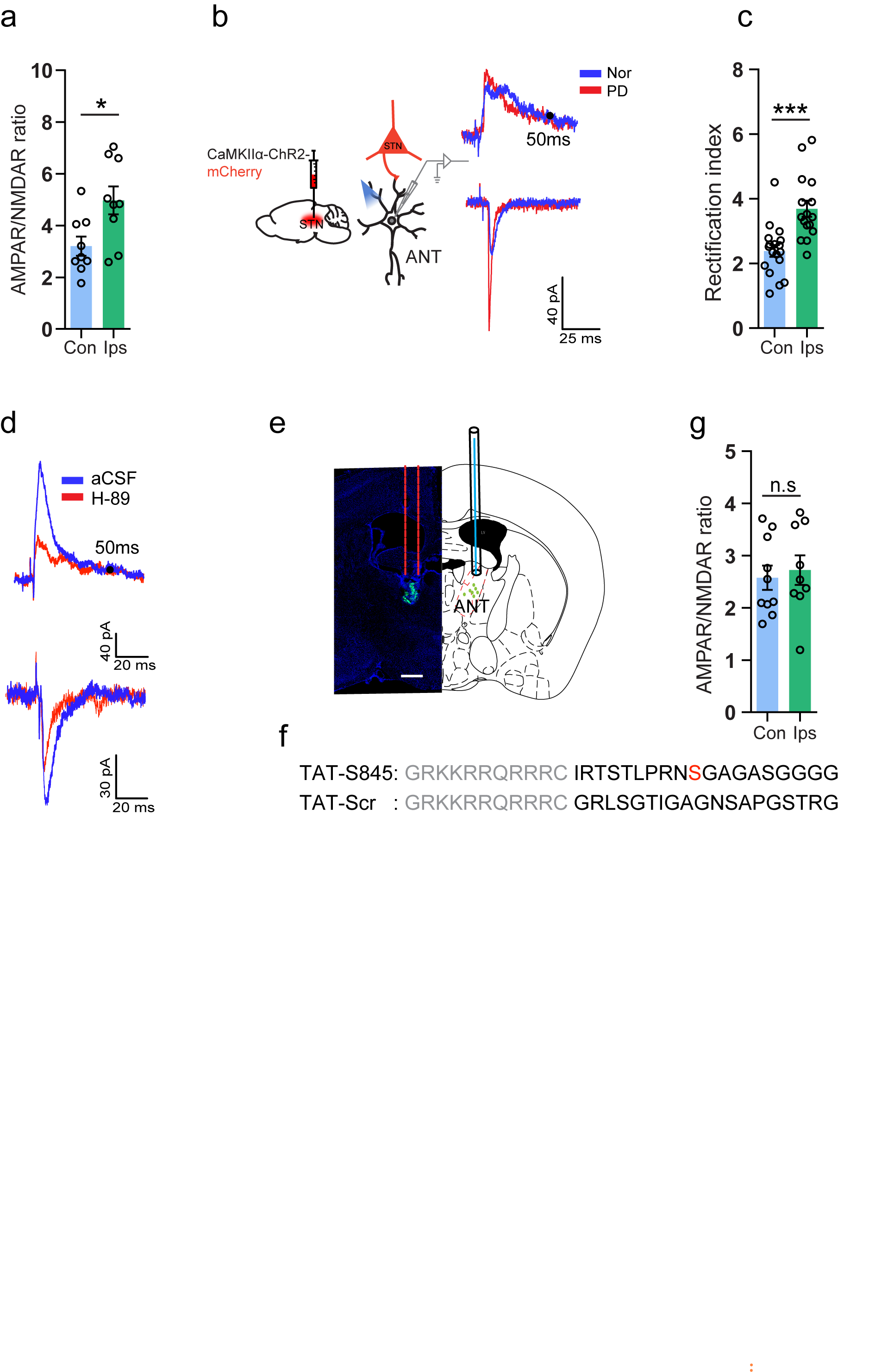


**Supplemental Fig 4 (Fig. S4). STN-ANT synaptic plasticity is crucial for PD motor deficits** a. Ratio of AMPAR/NMDAR currents evoked by electrical stimulation in contralateral and ipsilateral ANT neurons of the PD mice. *n* = 4-6 mice in each group. b. schematics of optical stimulation of STN projection fibers combined with whole cell recording on ANT slices (left), representative trace of evoked AMPAR currents holding at -70 mV and evoked NMDAR currents holding at +40 mV of normal mice (blue) or PD mice (red)(right). c. Rectification index evoked by electrical stimulation in contralateral and ipsilateral ANT neurons of the PD mice. *n* = 4-6 mice in each group. d. Representative trace of evoked AMPAR currents by electrical stimulation holding at -70 mV and evoked NMDAR currents holding at +40 mV in the presence of aCSF (blue) or H-89 (red) (right). e. Representative infusion site verified by fluorescent beads injected (1 μl) into ipsilateral ANT. Scale bar, 500 μm. f. The sequence of TAT-S845 and its scramble (scr). g. The ratio of AMPAR/NMDAR current evoked by electrical stimulation in contralateral and ipsilateral GPi neurons. *n* = 7 mice. Each circle in all panels represents a neuron. Data are mean ± SEM. **P* < 0.05; ****P* < 0.001; n.s, no significance.
